# Gastrıc cancer surgery in elderly patients: promising results from a mid-western population

**DOI:** 10.1186/s12877-023-04206-4

**Published:** 2023-08-30

**Authors:** Hilmi Yazici, Ahmet Cem Esmer, Ayse Eren Kayaci, Sevket Cumhur Yegen

**Affiliations:** Pendik Research and Treatment Hospital, General Surgery Department, Marmara University, Istanbul, Turkey

**Keywords:** Cancer, Elderly, Gastric, Surgery, Complication

## Abstract

**Backgrounds:**

Extended resection for gastric cancer in elderly patients is still challenging for surgeons. This study aimed to evaluate the prognosis and the postoperative outcomes of elderly patients underwent gastric cancer surgery in a high-volume center.

**Methods:**

The medical records of patients with gastric cancer surgery at Marmara University Hospital’s General Surgery Department were examined retrospectively. Patients were divided into two groups: Age ≤ 70 and Age > 70. The clinicopathological data of the patients were compared. The prognostic factors regarding gastric cancer surgery were analyzed with Cox proportional regression models. Kaplan Meier analysis and log-rank test were used to compare Overall Survival (OS) and Cancer-Specific Survival (CSS) among the groups. Competing risk regression analysis was used to examine cause-specific hazards among elderly patients.

**Results:**

The number of eligible patients was 250. Age > 70 group was 68 patients, and Age ≤ 70 group was 182 patients. There is no significant difference between the patient’s demographics or pathological outcomes. Neoadjuvant therapies performed less in elderly patients [40 (22%) vs. 7 (10%), p: 0.03, respectively]. There was no significant difference in severe complication (≥ Grade III) rates in both groups. Multivariate analysis showed that advanced T stage and adjacent organ invasion were the independent risk factors for OS. No significant difference was observed between the groups regarding OS (Log Rank (Mantel-Cox): 0.102). Younger patients have worse CSS than those who are older. Cause-specific hazard model demonstrated a not increased hazard ratio [HR: 1.04(0.78–1.38)] for elderly patients for OS and CSS.

**Conclusion:**

Gastric resections can be safely performed for elderly patients diagnosed with gastric cancer. This study showed that growing age is no longer a factor that will affect the clinician’s decision in performing surgery in gastric cancer patients.

## Introduction

Gastric cancers are the fifth most common malignancy worldwide and the third in cancer-related deaths, according to the latest data [1]. In addition, gastric cancers have had rising incidence trends in recent years [2]. In recent studies, the elderly population rate has been increasing worldwide, especially in the last two decades [3]. As a result of this, cancer incidence in the geriatric population is growing [4].

Surgery is still the only curative treatment for gastric cancer patients. Locally advanced lesions and pre-operative lymph node metastasis should be considered for neoadjuvant therapy. After detecting the tumor’s resectability, extensive surgery, including regional lymph nodes, should be performed on the patients [5]. Elderly patients also have comorbid diseases more common; moreover, the physiological reserves of almost all organ systems are reduced. Therefore, extensive oncological resections remain controversial in this population [6]. For most of the trials in this area, elderly patients are usually excluded from the studies [7]. Furthermore, studies on these procedures’ outcomes are limited.

The main objective of this study is to evaluate the perioperative results of both younger and elderly populations and discuss the safety of gastric resections on geriatric patients. The second objective is to review clinicopathological outcomes from the surgical oncology perspective.

## Material-method

Data regarding patients underwent gastric cancer surgery between January 2018- December 2020 in the Marmara University Hospital’s General Surgery Department were retrospectively analyzed. Patients who underwent surgery with gastric adenocancer but could not perform curative surgery were excluded from the study. A total number of 250 patients were included in the study. Patients were divided into two groups according to the age 70.

The patients’ clinicopathological data were retrospectively obtained by reviewing their medical records and operative reports, and the short- and long-term outcomes were analyzed. Surgical procedures were assessed according to the Japanese gastric cancer treatment guidelines 5th English edition [5]. Postoperative complications were evaluated according to the Clavien-Dindo classification, and grade III and higher complications, which described requiring surgical, endoscopic, or radiological intervention, were included in the analysis [8].

Pathological outcomes of the patients were evaluated according to The Eighth Edition AJCC Cancer Staging Manual [9]. Pathological responses to the neoadjuvant therapy were examined according to the College of American Pathologists Protocol for the Examination of Specimens From Patients With Carcinoma of the Stomach [10].

Following surgery, all patients were observed every three months for the first two years, every six months for the next five years, and then yearly until they passed away. Every appointment included a physical examination, laboratory tests, scans, and endoscopy. From the day of the surgical resection to the time of death or the last follow-up, the overall survival (OS) rate was calculated. Subsequently, the patients who were dead because of other causes (DOC) (cardiovascular, respiratory, infectious, neurogenic etc.) were noted, and Cancer-Specific Survival (CSS) was analyzed according to the competing risk regression analysis and cause-specific hazard models.

This study was approved by the Ethics Committee (No: 22.07.2022.995).

The primary outcome of this study is to evaluate the prognosis of operated gastric cancer patients according to the age of 70.

The secondary outcomes of the study are to compare the surgical and pathological results of the two groups and to analyze the differences in postoperative morbidity between the two groups.

### Statistical analyze

SPSS version 24.0 (Spss inc. IBM, Chicago, US) was used for statistical analysis. The proportion or frequency was compared between the two groups using Fisher’s exact test or the χ2 test, and differences in continuous variables were evaluated using the Student’s T-test and the Mann-Whitney U test for non-parametric values. Independent prognostic factors were identified by Cox proportional hazards regression model. Survival curves were estimated using the Kaplan-Meier method and compared using the log-rank test.

### Competing risk analysis

The R packages “cmprsk”, “tidycmprsk”, “ggsurvfit”, and “ggtsummary” were used in the paper. These packages are widely utilized tools for supporting analytical processes such as survival analysis, graph generation, and result reporting. The “cmprsk” package is employed for analyzing time-to-event data related to survival analysis, cumulative incidence function, and recurrence analysis. The “tidycmprsk” package works in conjunction with the “cmprsk” package to facilitate the organization and visualization of results, presenting the outputs of the “cmprsk” package in a more comprehensible manner. The “ggsurvfit” package provides auxiliary functions for visualizing Kaplan-Meier estimates used in survival analysis. Survival curves can be generated as graphs to compare different groups or variables. The “ggtsummary” package is used to visualize and report the results of Cox regression models employed in survival analysis. Summary statistics, tables, and graphs can be presented to summarize the outcomes of regression analyses. These packages represent the tools commonly used for data analysis and result visualization in the context of the paper.

## Results

Between January 2018-December 2020, 250 gastric cancer surgery were performed in the general surgery department. The patients were examined in two groups: <Age 70 and > Age 70. There were 68 patients in the > Age 70 group and 182 in the < Age 70 group.

Patient demographics and basic laboratory analysis are shown in Table 1. There was no significant difference in age, gender, operation type, postoperative hospital stay, and combined organ resections. Comorbid diseases are significantly more common among elderly patients [Respectively, 100 (55%) vs. 53 (78%), (p: 0.001)]. Neoadjuvant therapy was significantly higher in the < Age 70 group [respectively, 21% (n: 40) vs. 10% (n: 7), p; 0.03]. Tumor markers were similar between the two groups. Although the mean hemoglobin value pre- and postoperative was significantly lower in the elderly patient group, no statistically significant difference was observed on the mean hemoglobin difference pre and post-operatively. Pre-operative albumin levels were lower in the elderly patient group [Respectively, 3.95(± 0.4) vs. 3,71(± 0.5), p: 0.003].

**Table 1:** Patient demographics and operative results in both age groups. DSG: Distal Subtotal Gastrectomy PSG: Pro ximal Subtotal Gastrectomy TG: Total Gastrectomy

Total N: 250 Median (IQR)-Mean (± SD)	≤Age 70 (N: 182)	>Age 70 (N: 68)	p
Gender			
Female	59	28	0,196
Male	123	40	
BMI(Kg/m^2^)	25.2(± 4,3)	24.6(± 4.1)	0,89
Neoadjuvant Therapy			
Yes	40	7	**0,030**
No	142	61	
Comorbid Diseases			
Yes	100	53	**0.001**
No	82	15	
Surgery Type			
DSG	78	32	0,232
PSG	7	4	
TG	95	29	
Others*	2	2	
Postoperative Hospital Time (Days)	5(1)	5(3)	0,822
Combined Organ Resection			
Yes	19	9	0,559
No	163	59	
Tumor Markers			
CEA(μg/L)	1.79 (2.24)	1.97(2.34)	0,215
CA 19 − 9(U/ml)	13.3(17.7)	10.8(23.7)	0,939
CA 125(U/ml)	8,2(5.8)	10.5(22,8)	0,846
Pre-Operative Hb(g/dL)	11.9(± 2,1)	11.1(± 2)	**0.037**
Post-Operative Hb(g/dL)	9,8(± 2.1)	9.4(± 1.5)	**0,046**
Difference in Hb	2(1.85)	1.9(2.55)	0.644
Pre-Operative Albumin(g/L)	3.95(± 0.4)	3,71(± 0.5)	**0,002**

(SD: Standart Deviation, IQR: InterQuartile Range)

*Two peritoneal carcinomatosis, two second surgery for DSG to TG

Table 2 shows the pathological outcomes of the patients. There was no statistically significant difference between the two groups in Stage T, Stage N, peritoneal dissemination, adjacent organ invasion, and lymphovascular invasion. Pathological responses to the neoadjuvant therapy were similar between the two groups.

**Table 2:** Pathological outcomes in both age groups

Total N: 250 Median (IQR)-Mean (± SD)	≤Age 70 (N: 182)	>Age 70 (N: 68)	p
Stage T	(N: 178)	(N: 66)	
T1	16	11	0.058
T2	15	1	
T3	58	15	
T4	89	39	
Stage N	(N: 180)	(N: 66)	
N0	46	22	0.648
N1	25	7	
N2	39	12	
N3	70	25	
Pathological Stage			
Full Response	3	0	0,305
Stage I	25	11	
Stage II	32	14	
Stage III	113	38	
Stage IV	9	5	
Lymphovascular Invasion	(N: 180)	(N: 66)	
Yes	148	52	0.342
No	32	14	
Adjacent Organ Invasion	(N: 180)	(N: 66)	
Yes	19	9	0,559
No	161	57	
Peritoneal Cytology	(N: 106)	(N: 33)	
Positive	24	13	0.057
Negative	82	20	
Responses to Neoadjuvant	(N: 40)	(N: 7)	
Complete Response	3	0	0.724
Near Complete Response	6	1	
Partial Response	10	3	
Poor or No Response	21	3	

(SD: Standard Deviation, IQR: InterQuartile Range)

Early postoperative complications were examined according to the Clavian-Dindo Classification (Table 3). In total, 27 patients (15%) in the < Age 70 group and 15 patients (22%) in the elderly group had grade III and higher complications, and there was no significant difference between the groups (p: 0.174). In addition, each complication was examined among the two groups. There was no significant difference between the incidences of severe complications separately.

**Table 3:** Perioperative Complications (30 days)

Total N: 250 Median (IQR)-Mean (± SD)	≤Age 70 (N: 182)	>Age 70 (N: 68)	p
Complication			
Intra-Abdominal Abscess	3	0	0.274
Intra-Abdominal Hematoma	0	1	0.102
Ileus	3	3	0.203
Ischemic Colitis	1	0	0.540
Anastomosis Leakage	5	2	0.934
Pancreatic Fistula	4	1	0.714
Stump Leakage	7	4	0.484
Chylous Fistula	1	1	0.466
Pulmonary Embolism	1	0	0.540
Mortality	2	1	0.810
Complication Grades*			
IIIA	20	8	
IIIB	4	4	
IVA	1	0	
IVB	0	2	
V	2	1	
≥ IIIA	27 (15%)	15 (22%)	0.174

(SD: Standart Deviation, BMI: Body Mass Index, IQR: InterQuartile Range)

* Grades according to the Clavian-Dindo Classification

Univariate analysis and Kaplan-Meier log-rank test were performed on the variables regarding OS. In the univariate analysis, Stage pT3-4, Stage N+, lymphovascular invasion, and adjacent organ invasion were found to be prognostic factors for OS. Then a multivariate Cox Regression analysis was performed with these significant variables. Stage pT3-T4 and adjacent organ invasion were independent prognostic factors in the multivariate Cox regression analysis (Respectively, p: 0.020 and p: 0.028). Even though Age > 70 was not significant in the Univariate Analysis, it was included in the Multivariate analysis. Nevertheless, Age > 70 years was not a prognostic indicator for operated gastric cancer patients. The univariate analysis and multivariate analysis of factors on the prognosis of gastric cancer were demonstrated in Table 4.

**Table 4:** Univariate and Multivariate Analysis on Overall Survival

N:250	Log-rank*	HR	95% CI	p	HR	95% CI	p
Gender	0.606	0.909	0.630–1.311	0.610			
Age70	0.102	1.369	0.934–2.006	0.107	0.685	0.457–1.027	0.067
Neoadjuvant	0.164	1.338	0.883–2028	0.170			
Comorbidity	0.208	1.263	0.874–1.826	0.213			
Pre-Operative Hb		0.990	0.914–1.074	0.814			
Difference in Hb		1.185	1.052–1.334	**0.005**	1.121	0.991–1.269	0.070
Albumin		0.753	0.511–1.108	0.150			
Stage T (T1-T2 vs. T3-T4)	**< 0.001**	4.371	2.133–8.957	**< 0.001**	0.356	0.149–0.851	**0.020**
Stage N (N0 vs. N+)	**0.005**	1.862	1.192–2.909	**0.006**	1.145	0.634–2.068	0.643
Lymphovascular Invasion	**0.001**	1.588	1.191–2.117	**0.002**	1.749	0.757–4.043	0.191
Adjacent Organ Invasion	**< 0.001**	0.383	0.238–0.615	**< 0.001**	1.836	1.066–3.161	**0.028**
Peritoneal Cytology(+) (N:139)	0.115	0.666	0.405–1.096	0.110			
Complication	0.131	0.726	0.477–1.107	0.137			

*Log-Rank tesl was performed on the categorical variables.

Hb: Hemoglobin

**Fig. 1:**
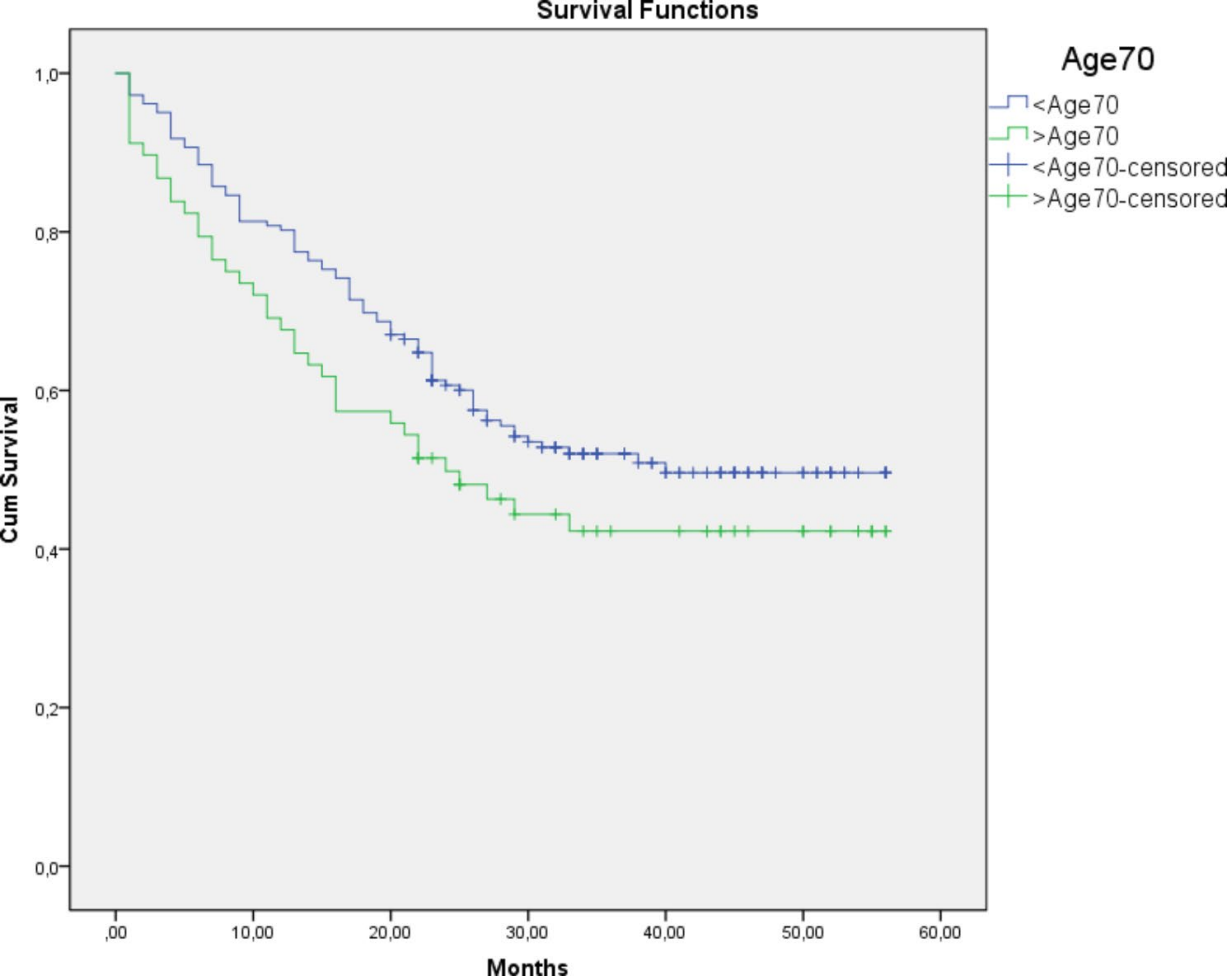
Overall Survival Analysis of both groups. Log Rank (Mantel-Cox): 0.102

**Fig. 2:**
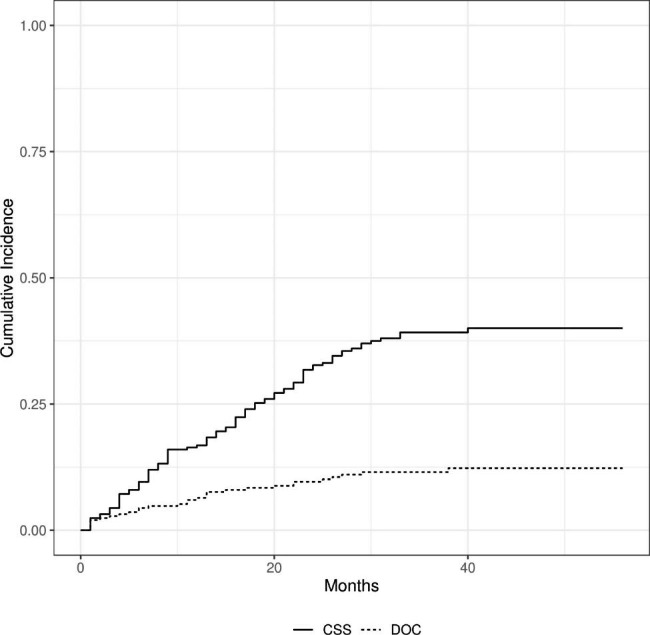
Cumulative Incidence Function of Cancer-Specific Survival and Death from Other Causes

**Fig. 3:**
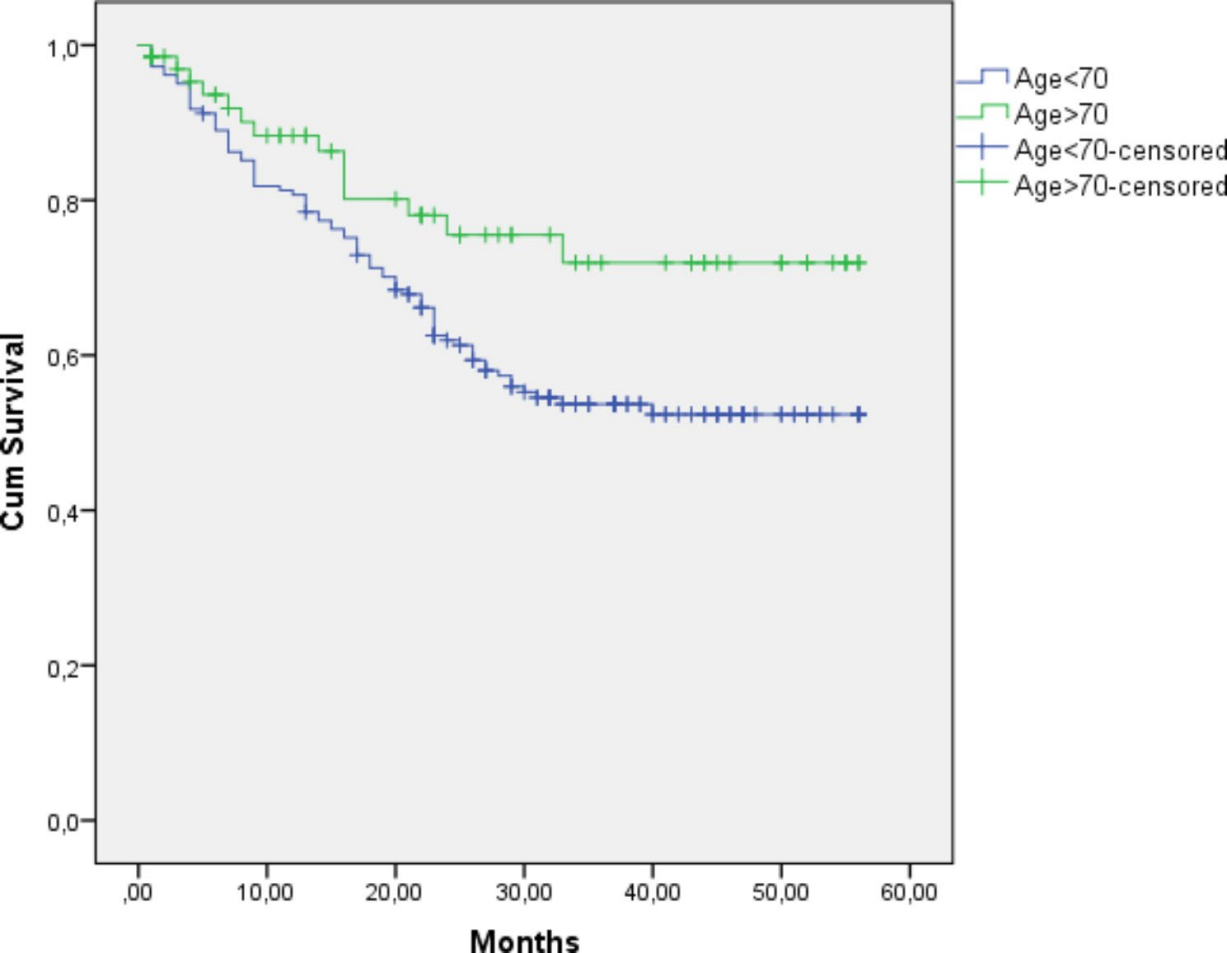
Cancer-Specific Survival Analysis Log Rank (Mantel-Cox): 0.138

**Fig. 4:**
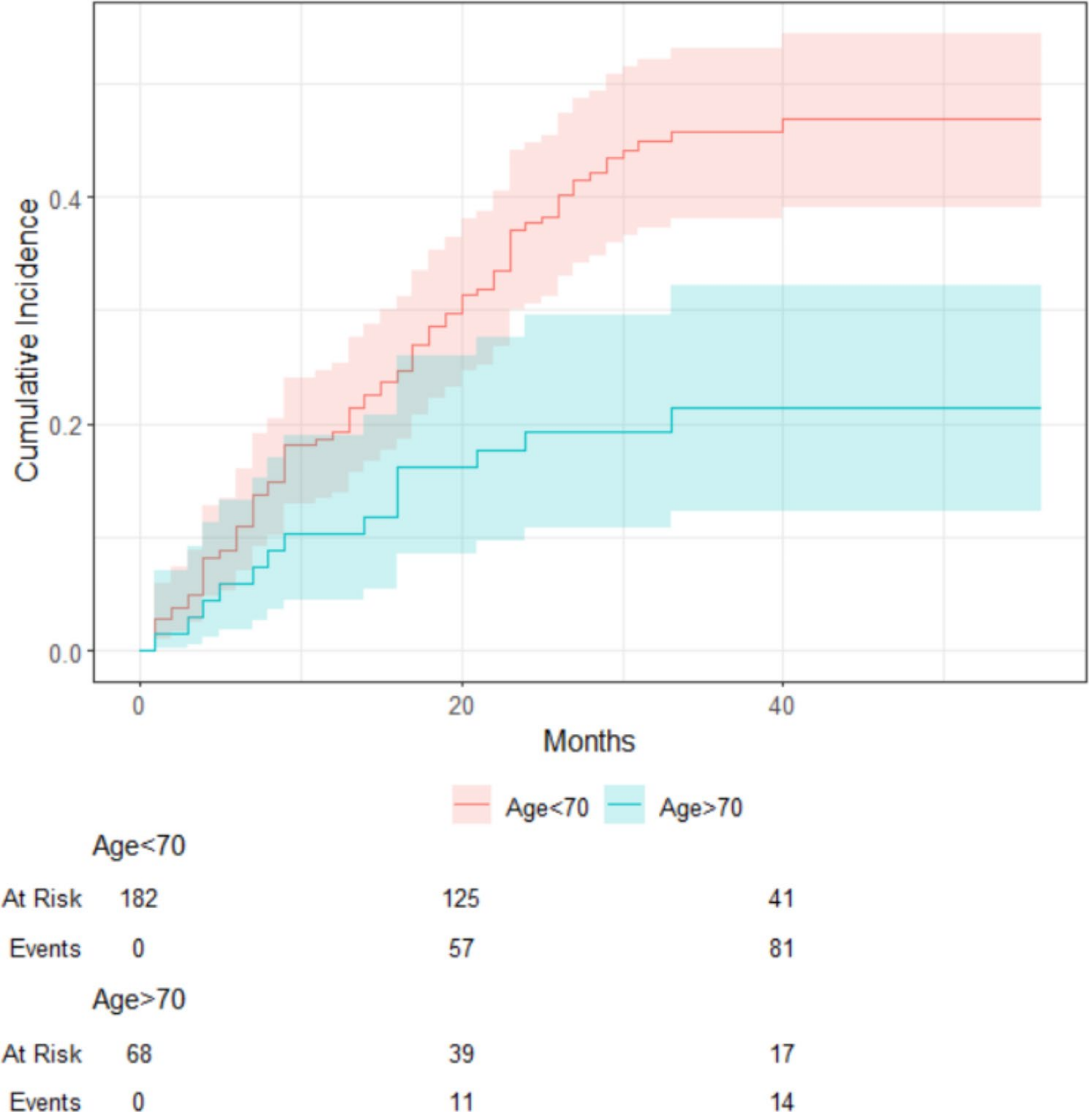
Cumulative incidences of Cancer-Specific Survival competing risks according to Age 70

The median follow-up of the cohort was 25 (24.2) months. The mean survival of all patients was 34.4 (± 1.4) months. Overall, the 5-year survival was 50.4% in all patients. The < Age 70 group demonstrated a higher 5-year OS rate (52.7%) (mean survival was 35.8 ± 1.6 months) compared to the Elderly group (44.1%) (mean survival was 30.6 ± 2.8 months). However, the difference in survival was not statistically significant (p = 0.102) (Fig. 1)., There were five patients (3%) in the younger group, and 24 (35%) patients in the elderly group died because of other causes (DOC) (p: <0.001) (Fig. 2). According to the competing risk analysis, DOC patients had not experienced gastric cancer-related mortality; hence, they should be accounted for in CSS analysis. Kaplan Meier analysis showed significantly shorter CSS in the younger patient group. (log-rank: 0.029)(Fig. 3). The cumulative incidence of both groups with competing risk analysis was summarized in Fig. 4. Gray’s test showed a significant difference in both groups regarding CSS (Table 5). Moreover, the Cause-Specific Hazard model showed no increased risk for OS and CSS in the elderly patients group (Table 6).

**Table 5:** Cumulative Incidence for competing risk

Characteristic	3-years Cumulative Inc.	P value*
Age 70		
Age < 70	46%(38% − 53%)	**< 0.001**
Age > 70	21%(12% − 32%)	

*Gray’s Test

**Table 6:** Competing Risk Regression Analysis HR: Hazard Ratio, CI: Confidence Interval

Characteristic	HR	95% CI	P value*
Age 70			
Age > 70	1.04	0.78–1.38	0.800

Cause-Specific Hazards

## Discussion

While the World Health Organization shared data on the elderly population, it included patients aged 60 and over [11]. To our best knowledge, there is no clear definition to describe this population’s thresholds. Current studies used different cut-off values for data regarding the elderly population [12–15]. According to the latest data from United Nations, the global life expectancy as of 2023 was 73.4 years. It might vary from region to region and country by country. It was preferred to choose a closer cut-off level to this average. Therefore, age 70 was used as a cut-off value in this study to determine the different age groups.

Aging, frailty, and surgical requirements are all rising sharply around the world, and surgeons are always struggling with the trade-off between immediate results and the effectiveness of surgery in the elderly. But recent research clearly indicates that age by itself does not predict the likelihood of problems in older individuals undergoing elective surgery, but cognitive or functional fragility does. Neither a referral doctor nor an evaluating surgeon should refuse to do surgery on a patient based only on their age. Decisions should instead be based on a CGA (comprehensive geriatric assessment) that provides a clear picture of the patient while taking into account their cognitive, functional, nutritional, socioeconomic, and emotional health [16]. Additionally, Recent studies examining the use of preparative CGA in surgical patients showed encouraging results on postoperative outcomes in old and/or frail patients [17].

This study has some limitations that need to be acknowledged. First is the retrospective design. Second, the study population is a cohort of gastric cancer patients treated in a single center for only three years. Therefore, results may not be generalizable to all patients undergoing gastric cancer surgery. Finally, procedure subgroups were not randomly assigned and differed in their tumor stages as well as their treatments.

The proportion of neoadjuvant therapies was significantly lower in the elderly patient group. It is consistent with existing studies. Nienhauser et al. reported the rate of patients treated with neoadjuvant therapy was constantly trending down with increasing age [18]. The effect of neoadjuvant therapies on elderly patients was less discussed in the current literature. Jiang et al. reported that older patients (> 60 years of age) had significantly higher pathological response rates to neoadjuvant therapy [19]. Contrary, Choi et al. reported that the cancer type, lymph node metastasis, and cancer stage did not differ significantly [20]. In this study, there was no significant difference between the two groups among pathological responses to neoadjuvant therapy. In addition, there was no statistical difference in all T, N, and M stages in both age groups. As a result, this issue still remains controversial in current studies. Further investigations are needed for this topic.

Perioperative complication rates are unclear in the previous studies. Some studies argued that the elderly population has an increased risk for complications after gastric cancer surgery [12, 21]. On the contrary, some recent studies reported the risk of complications was similar in the older patient population [22]. Gretschel et al. reported that there was no significant difference in surgical complications in their study; however, the same study showed a significant increase in-hospital mortality [23]. Wakahara et al. reported that perioperative blood loss might increase surgical complications [14]. In the previous study, although there was a statistically significant difference between pre-and postoperative mean hemoglobin levels, no significant difference was found in total blood loss. Therefore, similar complication rates between the two groups in this study might be a consequence of this. Several studies have reported that pre-operative albumin levels might strongly predict postoperative complications [24, 25]. In Kang et al. study, the authors declared that pre-operative decreased albumin levels might be a reflection of malnutrition [25]. In this study, mean albumin levels in both groups are in the normal physiological range. Although the difference in albumin levels is significant, it was not supported by the rates of complications.

There are many studies reported that elderly patients have a poorer prognosis than those who are younger [15, 26, 27]. Liang et al. reported that elderly patients had worse prognosis than the younger [15]. They also noted that CSS rates were similar among them. Kauppila et al. showed that the Hazard Ratios of 5-year all-cause mortality increased after age 70 [28]. In SWEGASS study, the authors reported that the adjusted Hazard Ratio was increased in patients of older age (< 65 vs. >75) [29]. They also mentioned that age was less likely to be a factor for CSS. However, there were only a few studies reported similar OS rates in elderly patients. Wakahara et al. performed a stage-matched prognostic analysis. There were no significant differences in the 5-year OS among elderly patients with Stage I, Stage II, and Stage III diseases [14]. In the previous study, DOC were seen significantly more frequently in the elderly patient group, as expected. This study is one of the few studies that performed competing risk analyses regarding CSS and DOC. After competing risk regression, Age > 70 group had comparable life expectations with those who are younger. In other words, the elderly patients who underwent gastric cancer surgery had comparable overall survival outcomes to younger patients. This was contrary to most of the existing literature.

The younger gastric cancer patients showed worse CSS in the present study. This might be explained primarily by tumors nature. In a large-scale study, Lu et al. found that younger patients with gastric carcinoma had poor histological types and worse prognoses [30]. In a review, Li reported that younger patients are more likely to be at an advanced stage, to have poor differentiation and, worse Borrman category [31]. The author also reported that elderly patients have comorbid diseases more commonly. This was consistent with our findings. The balance between these two findings might explain the OS similarity and life expectations between the two groups.

Adjuvant chemotherapy after definitive surgery is one of the most important factors in the survival outcomes of gastric cancer patients. The CLASSIC trial demonstrated improved 5-year disease-free survival among patients > 65 years of age [32]. However, the elderly population is taking less aggressive chemotherapy regimens due to decreased physiological and organ status. Hence, keeping the balance between the benefits of chemotherapy and its side effects is essential for clinicians.

## Conclusion

After the latest updates and improved techniques, surgical treatment for gastric cancer is safe and feasible in the elderly patient group. In selected patients, combined surgery with appropriate neo/adjuvant therapies has comparable short- and long-term results with younger age groups.

## Data Availability

The datasets used and/or analysed during the current study are available from the corresponding author on reasonable request.
